# Sexuality and contraceptive knowledge in university students: instrument development and psychometric analysis using item response theory

**DOI:** 10.1186/s12978-019-0791-9

**Published:** 2019-08-22

**Authors:** Sebastian Sanz-Martos, Isabel M. López-Medina, Cristina Álvarez-García, Carmen Álvarez-Nieto

**Affiliations:** 1 Research Group Nursing and Healthcare Innovation (CuiDsalud), University of Jaén, Jaén, Spain; 2 Department of Nursing. Faculty of Health Sciences, Research Group Nursing and Healthcare Innovation (CuiDsalud), University of Jaén (Spain), Edif. B3, Dep. 265, Campus Las Lagunillas, s/n, 23071 Jaén, Spain

**Keywords:** Validation studies, Questionnaires, Knowledge, Young adult, Contraception

## Abstract

**Background:**

As a consequence of biological, psychological and social changes during puberty, youth is a period characterized by impulsiveness and risk-taking. Members of this population often feel invulnerable and have a strong motivation to explore their identity. A good level of knowledge is necessary to allow young people to experience their sexuality in a healthy way, without associated risks. In our environment there is currently no valid Spanish-language tool to measure the level of knowledge about sexuality and contraception. This study sought to develop and test the psychometric properties of a new sexuality and contraception knowledge instrument.

**Methods:**

This is a cross-sectional study to validate the sexuality and contraception knowledge instrument. The validation process followed four phases: (1) development of the instrument, (2) content validation by an expert panel, (3) pilot test and (4) psychometric analysis of the instrument using item response theory according to the Rasch model. The validation process took place from September 2017 to February 2018.

**Results:**

The sample included 387 students enrolled at the Nursing and Law degrees from the University of Jaen. The final instrument was made up of 15 items. All of the items presented good adaptation values with respect to the model. The scale showed good fit and reliability: 0.99 for items and 0.74 for people. The temporal stability of the scale was calculated using test–retest, obtaining a value of 0.81 (CI 0.692–0.888). The construct validity showed the one-dimensionality of the construct, while the discriminant validity obtained good results, so the scale appears to be able to differentiate between participants with low or high levels of knowledge.

**Conclusion:**

The results suggest the Sexuality and Contraception Knowledge Instrument is psychometrically valid and reliable for measuring the knowledge level concerning sexuality and contraceptive methods in young university students.

## Plain English summary

Youth is a period characterized by impulsiveness and risk-taking, which make it a risky age period for unwanted pregnancy. To explore their sexuality in a healthy way, without associated risks, it is necessary for youths to have a good level of knowledge. In this study, we developed and tested the psychometrical properties of a scale to measure the sexuality and contraceptive knowledge of young students at a Spanish university.

In this paper we evaluate the psychometric properties of a scale to measure the level of knowledge about sexuality and contraceptive methods in young university students.

The sample included one hundred and eighty-seven students enrolled at the Nursing and Law degrees from the University of Jaen. The final version of the scale showed good adaptation values with respect to the Rasch model and reliability for the items and the persons. The scale obtains good results for discriminant validity, so it appears to be able to differentiate between participants with low or high levels of knowledge. Finally, the scale showed good values of temporal stability.

So, the final version of the scale is valid and reliable to measure the knowledge level on sexuality and contraceptive methods in university youth students.

## Introduction

Unwanted pregnancies are still a public health problem in developed countries. It is estimated that more than half of all unintended pregnancies occur through misuse or lack of continuous use of contraceptive methods [1–3]. Spain had a fertility rate for the 15–19 age group of 7.04 pregnancies per 1000 women and 25.23 pregnancies per 1000 women for the 20–24 age group in 2017 [4].

The youth population is defined as those aged between 15 and 24 years [5]. Youth is a period of age characterized by impulsivity and risk-taking: young people feel invulnerable. This, combined with the search for their own identity, entails a need for experimentation that conditions early sexual initiation, with the consequent risk of unwanted pregnancies, abortions and sexually transmitted infections [5, 6].

Pregnancies that happen unintentionally place the mother at risk. From the moment the young person learns she has become pregnant, she is susceptible to the onset of psychological disorders such as anxiety, feelings of desperation, feelings of hopelessness and feelings of guilt for having failed in her family environment, to which is sometimes added the dissolution of the couple and/or the man’s refusal to assume paternity. Many young women have to drop out of school, which limits their future employment opportunities. She sees her youth abruptly cut short and is suddenly immersed in an adult world, without an adult’s psychological maturity, which is sometimes exacerbated by the loss of her family and an emotionally supportive environment [7, 8].

The feelings and emotions that accompany sexuality are diverse, marked by the myths that they evoke from their infancy, reinforced by disinformation and shame acquired when trying to obtain information about the questions that arise and that allow them to explore sexuality in a health fashion [5, 9].

Related to the concept of healthy sexuality, the concept of sexual and reproductive health is defined as a general state of physical, mental and social well-being and not the mere absence of disease or ailments in all aspects related to the reproductive system, its functions and processes [10]. This definition implies a positive approach to living a healthy and risk-free sexuality that allows one to enjoy a satisfying sexual life. As a consequence of sexual awakening and all of the matters related to the experience of sexuality, young people need to have solid knowledge to be able to find the answers to all the questions they may have [11]. The main barriers found among young people to the use of any contraceptive method are the difficulty in obtaining them [12] and the lack of knowledge about the different contraceptive options available [12–15].

A knowledge construct is defined as a set of ideas, concepts or experiences acquired through the senses that allow a group or individual to reach a higher level of reason. Adequate knowledge about sexuality and contraception is defined as the possession of training about sexuality and contraception that allows one to make informed decisions and pursue one’s sexuality safely [11].

In previous research with a university population, increasing the level of knowledge about sexuality and contraception has been considered a key element in the prevention of unwanted pregnancy [8, 15–21]. To measure this level of knowledge to gauge the effectiveness of educational programmes, ad-hoc tools have been used, which have lacked a validation process to guarantee their validity and reliability.

Scales used to measure a construct, must be valid and reliable. Validity is defined as the ability to measure what you really want to measure and not another dimension of the studied construct and reliability as the ability for the measurement to be consistent and accurate [22]. There are several types of validity that globally allow to check if the evaluated scale is valid to measure the construct [22]: a) content validity: the ability of the items that make up the scale to adequately evaluate the universe of all the dimensions that make up the study construct. b) construct validity: degree of agreement between what the study instrument evaluates and what it would theoretically be expected to measure. c) criterion validity: evaluates the relationship between the score of the evaluated scale and an external criterion that is usually another validated scale that measures the same construct.

Reliability consists of several components [23]: a) stability: evaluates the stability of the scores, which depends only on the level of mastery of the construct and not on the way in which the scale is administered or completed. b) internal consistency: The degree to which items measure the same criterion. c) interobservers reliability: degree of awareness of the scores when they are measured by two different people.

To live sexuality in a healthy way, it is necessary that youth have a high level of knowledge about sexuality and contraceptive methods and to measure that is necessary a valid and reliable tool. A valid Spanish-language tool to measure the level of knowledge about sexuality and contraception, would allow us to identify the main knowledge gaps remaining in educational programmes and so adapt them to youth educational needs. Given the lack of a tool to measure this level of knowledge about sexuality and contraception in young people, the creation and validation of a scale for knowledge about sexuality and contraceptive methods is justified. The purpose of the study was to develop and test the psychometric properties of a sexuality and contraception knowledge instrument (SexContraKnow-Instrument).

## Methods

### Study setting

A cross-sectional study was conducted to validate the SexContraKnow-Instrument using the item response theory. The sample consisted of students pursuing either a Nursing or Law degree from the university of Jaen. As inclusion criteria, participants were required to be between 18 and 25 years of age and to sign the informed consent form prior to the completion of the instrument. Participants who did not meet all of the inclusion criteria were removed.

### Design of the questionnaire

A literature review was carried out to develop the items that formed the first version of the instrument. The databases consulted were PubMed, CINAHL, Scopus, Cuiden Plus, LILACS and the IME database. The search covered publications until June 2017, using the search terms ‘unplanned pregnancy’, ‘primary prevention’ and ‘questionnaires and surveys’. We found 7 articles that use a scale to measure the knowledge level concerning sexuality and contraception in university students [24–30]. An initial bank of 24 items was formed, divided into two theoretical categories: knowledge about sexuality and knowledge about contraceptive methods. All items were written with statements that participants should answer as either ‘true’ or ‘false’, along with a third answer option, ‘don’t know/no answer’. A seven-person national expert panel, with previous experience on the construct, was consulted for their opinion about the sufficiency of the items generated from the bibliographic review to measure the construct. As result of this consultation, 13 new items were generated, forming the first version of the scale with 37 items. The consultations were made by email with a template of all items. Experts had to indicate the relevance of each item to measure the construct and in a final dialog box they had to add items not presented but necessary to measure the construct, as well as any suggestions they wanted to make.

### Content validation

The same seven-person national expert panel from the development of the scale phase were asked to evaluate the clarity and relevance of the items that formed the first version of the scale, using a Likert-type scale (1–5). In order to consult the expert panel, we conduct an expert judgment using the individual aggregates method. The degree of agreement among the experts was calculated using the Aiken statistic V, with a higher value of 0.7 for relevance and clarity of the item to be part of the instrument, and a value greater than 0.6 for the lower limit of its 95% confidence interval [31]. As result, 21 items were redrafted and 8 items were deleted as irrelevant for measuring the construct (Table 1). The resulting second version of the SexContraKnow-Instrument included 29 items.

**Table 1:** Degree of agreement among the expert panel

Item	Relevance Aiken’s statistic V	CI 95%	Clarity Aiken’s statistic V	CI 95%
1^b^	0.89	0.72–0.96	1	0.88–1
2^b^	0.82	0.64–0.92	0.93	0.73–0.98
3^b^	0.93	0.73–0.98	1	0.88–1
4^b^	0.93	0.73–0.98	0.82	0.64–0.92
5^b^	0.82	0.64–0.92	1	0.88–1
6	0.82	0.64–0.92	0.93	0.73–0.98
7^b^	0.82	0.64–0.92	0.96	0.82–0.99
8^b^	0.96	0.82–0.99	0.96	0.82–0.99
9^b^	0.96	0.82–0.99	0.96	0.82–0.99
10	0.93	0.73–0.98	0.86	0.69–0.94
11^b^	0.82	0.64–0.92	1	0.88–1
12^a^	0.79	0.60–0.90	–	–
13^b^	0.86	0.69–094	0.93	0.73–0.98
14^a^	0.68	0.49–0.82	–	–
15	0.93	0.73–0.98	0.93	0.73–0.98
16^b^	0.86	0.69–0.94	1	0.88–1
17^a^	0.54	0.36–070	–	–
18	0.89	0.72–0.96	0.96	0.82–0.99
19	0.96	0.82–0.99	1	0.88–1
20^a^	0.75	0.57–0.87	–	–
21^a^	0.61	0.42–0.76	–	–
22^a^	0.68	0.49–0.82	–	–
23^a^	0.68	0.49–0.82	–	–
24^a^	0.71	0.43–0.85	–	–
25^b^	1	0.88–1	0.96	0.82–0.99
26^b^	0.96	0.82–0.99	0.82	0.64–0.92
27	1	0.88–1	0.96	0.82–0.99
28^b^	1	0.88–1	0.96	0.82–0.99
29^b^	1	0.88–1	0.93	0.73–0.98
30^b^	1	0.88–1	0.93	0.73–0.98
31^b^	1	0.88–1	0.86	0.69–0.94
32^b^	1	0.88–1	0.96	0.82–0.99
33^b^	1	0.88–1	0.96	0.82–0.99
34	1	0.88–1	0.96	0.82–0.99
35^b^	1	0.88–1	0.96	0.82–0.99
36	1	0.88–1	1	0.88–1
37	1	0.88–1	1	0.88–1

^a^Deleted items

^b^Selected items with modifications

### Pilot test

For the selection of the pilot test sample, we used two-stage multistage sampling. In the first stage, 30 participants were selected through a random process among the attendees in each of the classes in the 4 courses of both grades. Subsequently, within each group, the questionnaires were numbered and through a simple random process, we selected 20 participants from each group (160 participants). The pilot test was conducted between September 11 and 22, 2017.

The clarity of the wording of the items was evaluated by a question with two answer options (yes/no), where we asked the participants to explain the reason if they indicated that they did not consider the item clear. The difficulty index, discrimination index and item-total correlation were calculated using the Pearson correlation coefficient. Items with negative correlations were reviewed and rewritten.

As a result, 13 items were eliminated because they did not exceed the critical value of 0.3 for the discrimination index and 6 were redrafted after the participant evaluation (Table 2). The resulting third version of the instrument consisted of 16 items.

**Table 2:** Item analysis

Item	Difficulty index	Discriminant index
1^a^	0.87	0.1
2^a^	0.99	0
3^a^	0.99	0
4^b^	0.84	0.1
5^a^	0.98	0.05
6^a^	0.99	0.025
7^a^	0.9	−0.05
8^b^	0.88	0.15
9^a^	0.98	0
10^a^	0.96	0.05
11^b^	0.18	0.33
13	0.19	0.25
15^b^	0.3	0.23
16^b^	0.26	0.43
18^a^	0.36	0.43
19	0.55	0.6
25^a^	0.83	0.1
26	0.58	0.43
27	0.34	0.3
28	0.61	0.58
29^b^	0.17	0.025
30	0.25	0.43
31	0.32	0.48
32	0.41	0.68
33	0.11	0.23
34	0.34	0.53
35^a^	0.11	0.1
36^a^	0.98	0
37^a^	0.80	0.1

^a^Deleted items

^b^Selected items with modifications

### Validation

For the validation of the scale, we followed the theory of response to the item and the Rasch model. The Rasch model [32] is ideal for determining the psychometric properties of a scale to measure the level of knowledge about sexuality and contraceptive methods, because it offers the advantage of measuring the level along a continuum of items with different levels of difficulty (from the easiest to the most difficult), which allows us to classify participants more accurately. Following this assumption, we established that the probability of guessing an item depended on the person’s ability level, considering this level of ability an invariable element, so that two people whose levels of ability are located in the same point of the variable continuum are assumed to present the same ability level of the study construct. The Rasch model offers the advantage of measuring the parameters of people and items and the objectivity of the test, because the differences in the ability level are due to the domain of the person and not to the items by which this ability is measured [33].

Prior to the realization of the Rasch model, we checked the assumptions of the model:

- Unidimensionality: The unidimensionality of the construct was evaluated by performing a parallel analysis [34] to determine the number of factors to be extracted; we compared the proper values presented by the data when performing an exploratory factorial analysis with those that would be obtained by chance according to the number of items and sample. The simulation was performed with Monte Carlo PCA® software [33].
- Local independence: We check the residual correlation matrix using Yen’s Q3, establishing critical values ±0.20 [35].
- Invariability: According to Rasch measurement theory, the scale should work in the same way, irrespective of which group is being assessed. If for some reason, one group does not display equal likelihood of confirming the item, the item would display Differential Item Functioning (DIF), which is an analysis of variance of the person–item deviation residuals in the analysis factor [36]. We tested the hypothesis by gender, academic degree and have received information about sexuality and contraception. The existence of differential item functioning indicates that subjects who have the same level of ability have a different probability of responding correctly to an item, which is related to bias, that is, favouring one group over the another in the evaluation by the scale
- Rasch’s model parameters were calculated for people and items. The values for the adaptation of the items to the model exceeded the values considered optimal (0.8–1.2), but, it appeared that all of the items forming the final version of the instrument have good values of adaptation to the model as they do not exceed the minimum acceptability values (0.5–1.5) [37]. The values of infit and outfit, in the Rasch model indicate how accurately or predictably data fit the model. The infit is an adjustment statistic with weighted information that focuses on the general behaviour of an item or a person. The outfit is an adjustment statistic sensitive to atypical cases, which allows you to determine unusual events that occur unexpectedly [37].

The difficulty index of the items was calculated using the Rasch model, where the value 0 was assigned to the average difficulty index.

Finally, the reliability of the scale was calculated by items and persons with the Rasch model, establishing a minimum acceptable value of 0.7. These analyses were carried out with the jMetrik program [38].

For the validation of the scale, a necessary sample of at least 300 participants was estimated [39]. Prior to the completion of the scale, all participants were asked to sign an informed consent form to voluntarily participate in the research. The initial sample included 421 participants, but 34 participants (20 from the Nursing degree and 14 from the Law degree programme) were eliminated because they were over 24 years old. The final sample consisted of 387 participants (208 from the Nursing degree and 179 Law degree). The completion time for the instrument was 10 min. Data collection occurred between September 27, 2017, and February 2, 2018.

### Statistical analysis

The comprehension validity of the instrument was calculated using the Flesch-Szigriszt formula, resulting in a value of 50.33 points (somewhat difficult). A semantic review of the items was carried out and the PMOSE/IKIRSCH formula was used to evaluate the organization of the tool’s content and density, obtaining a score of 6 points (low complexity). Both scores indicate that the instrument is suitable for the target population. All analyses were performed using the INFLESZ v1.0 program [40].

Construct validity was tested through exploratory factorial analysis with the final version of the instrument. A parallel analysis was carried out by a simulation to determine the number of factors to be extracted. Factors were extracted using principal components, and an orthogonal rotation was applied (Varimax, Equamax and Quartimax), selecting the rotation solution that gave us the best results.

The discriminant validity of the scale was tested with a hypothesis in known groups (comparing participants we theorized as having a greater mastery of the construct with those who did not). For this purpose, we compared the level of knowledge according to the academic course, the participants of the third and fourth courses of the Nursing degree having received formal training on the construct before, while the other groups had not received it (first and second course of Nursing Degree and the four courses of the Law Degree).

Temporal stability was assessed using the test–retest procedure, estimating the Intraclass Correlation Coefficient (ICC) and its 95% confidence interval. A group of 62 students from the second course of the Nursing degree completed the final version of the questionnaire twice over a two-week interval.

Finally, for the final 15-item version of the SexContraKnow-Instrument scale, we calculated the percentage of success, errors and ‘don’t know/no answer’ answers.

Data were analysed using SPSS 24.0 and jMetrik 4.1. There were no missing data.

## Results

Table 3 shows the main characteristics of the sample.

**Table 3:** Characteristics of the sample (*n* = 387)

Characteristics	Nursing Degree (*n* = 208)	Law Degree (*n* = 179)
Gender		
Male	38 (18.3%)	75 (41.9%)
Female	170 (81.7%)	104 (58.1%)
Age*	20.28 (1.789)	20.27 (1.641)
Academic course		
1st	64 (30.8%)	49 (27.4%)
2nd	47 (22.6%)	40 (22.3%)
3rd	36 (17.3%)	43 (24%)
4th	61 (29.3%)	47 (26.3%)
First intercourse		
Yes	176 (84.6%)	153 (85.5%)
No	32 (15.4%)	26 (14.5%)
Age of first intercourse*	16.87 (1.418)	16.78 (1.208)

Data expressed by frequencies and percentages, *mean and standard deviation

### Rasch model

For the 16 items version of the scale, the Kaiser–Meyer–Olkin (KMO) test showed a value of 0.773, and the Bartlett’s sphericity test obtained a significance value lower than the significance level 0.05, both indicating the adequacy of the data for factoring. Exploratory factorial analysis together with Horn’s parallel analysis showed that the construct is one-dimensional, finding that the own value for the second factor (1.264) is lower than that obtained in the simulation (1.286).

**Fig. 1:**
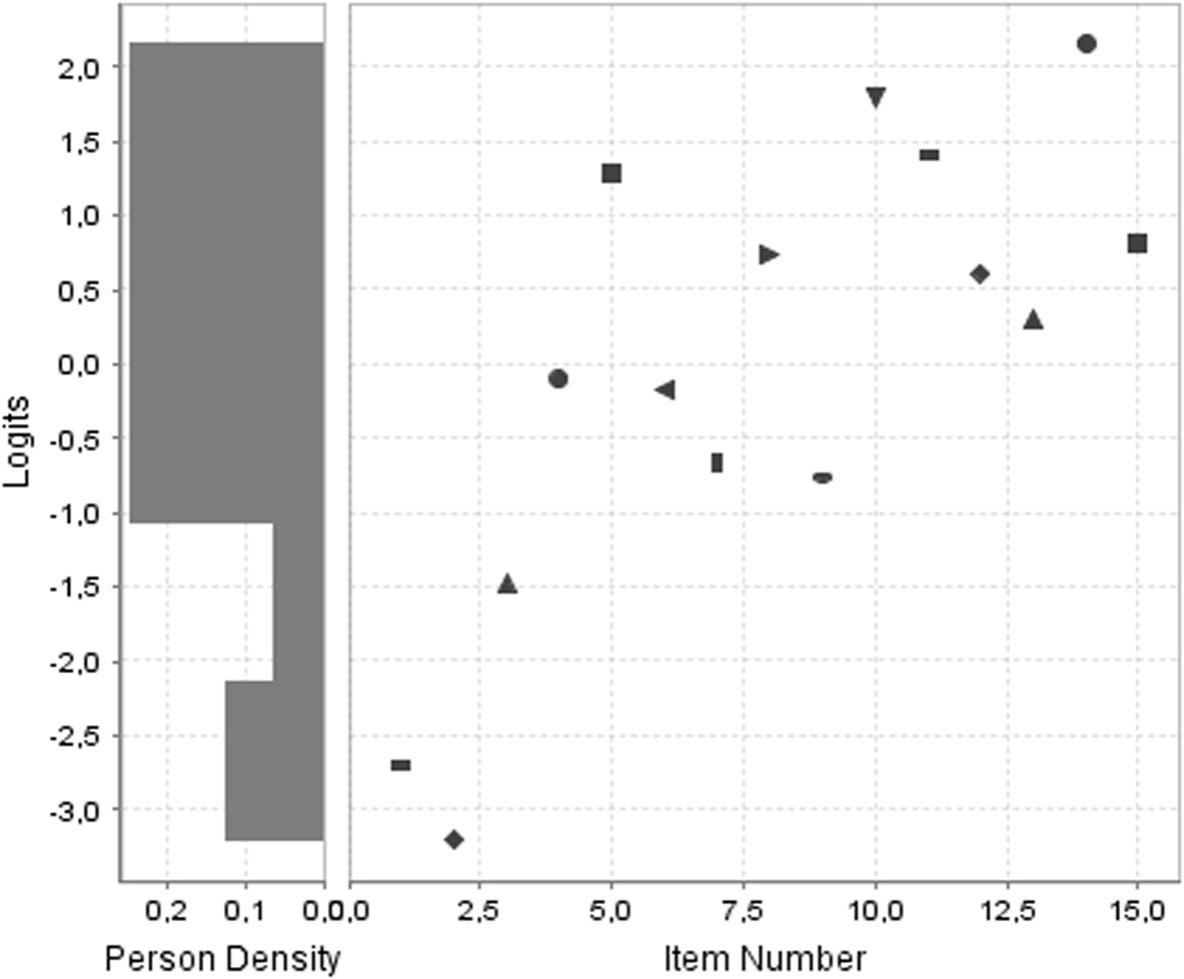
Item map for the final version of the scale

The first assumption for applying the Rasch model was verified, so it was possible to apply the Rasch model for the 16 items that formed the scale. In the Rasch model, item 11 presented higher values (1.77) regarding the outfit of the model, so we proceeded to eliminate it. In Table 4, we can see the adaptation values with respect to the Rasch model for the items after the elimination of item 11. The remaining 15 items presented good adaptation values with respect to the model, forming the final version of the instrument. The items showed central values at 1 for infit and outfit, verifying one-dimensionality. In Fig. 1 we can see the item map with the level of ability of the participants with respect to the level of difficulty of the items.

**Table 4:** Rasch model parameters

Item	Difficulty index	Standard error	Infit	Outfit
4	−2.77	0.18	1.14	1.29
8	−3.22	0.21	0.98	1.15
13	−1.44	0.13	1.12	1.38
15	−0.11	0.12	1.2	1.14
16	1.29	0.13	1.18	1.14
19	−0.18	0.12	1.03	1
26	−0.68	0.12	1.07	1.15
27	0.71	0.12	0.95	1.04
28	−0.69	0.12	0.83	0.75
29	1.70	0.14	1	1.01
30	1.41	0.13	0.84	0.66
31	0.57	0.12	0.92	0.83
32	0.34	0.12	0.87	0.89
33	2.23	0.16	0.89	0.68
34	0.83	0.12	0.98	0.88

Regarding local independence, the maximum value was obtained for items 30 and 31 with 0.27; the remaining items did not exceed the critical values of ±0.20.

The invariability of the 15 items of the scale was assessed for DIF across gender (male/female), formation (yes/no) and academic degree (nursing/law) (Table 5). Various significant DIF was found on the items of the final version of the scale; however, using the Bonferroni-adjusted alpha value (0.05/15 = 0.0033), the value become non-significant, so the 15 items of the scale were considered invariable.

**Table 5:** DIF analysis

Item	Gender	Academic degree	Formation
4	χ^2^ = 0.91; *P* = 0.34	χ^2^ = 0.02; *P* = 0.88	χ^2^ = 0.12; *P* = 0.73
8	χ^2^ = 0.44; *P* = 0.51	χ^2^ = 1.12; *P* = 0.29	χ^2^ = 0.25; *P* = 0.62
13	χ^2^ = 4.35; *P* = 0.04	χ^2^ = 1.12; *P* = 0.29	χ^2^ = 0.84; *P* = 0.36
15	χ^2^ = 0.11; *P* = 0.73	χ^2^ = 4.78; *P* = 0.03	χ^2^ = 2.64; *P* = 0.10
16	χ^2^ = 1.89; *P* = 0.17	χ^2^ = 0.03; *P* = 0.86	χ^2^ = 3.11; *P* = 0.08
19	χ^2^ = 2.26; *P* = 0.13	χ^2^ = 1.27; *P* = 0.26	χ^2^ = 0.41; *P* = 0.52
26	χ^2^ = 1.79; *P* = 0.18	χ^2^ = 0.73; *P* = 0.39	χ^2^ = 3.12; *P* = 0.08
27	χ^2^ = 3.17; *P* = 0.08	χ^2^ = 5.67; *P* = 0.02	χ^2^ = 1.09; *P* = 0.30
28	χ^2^ = 0.83; *P* = 0.36	χ^2^ = 5.41; *P* = 0.02	χ^2^ = 0.00; *P* = 1
29	χ^2^ = 0.02; *P* = 0.90	χ^2^ = 1.20; *P* = 0.27	χ^2^ = 2.00; *P* = 0.16
30	χ^2^ = 7.11; *P* = 0.01	χ^2^ = 0.25; *P* = 0.62	χ^2^ = 0.82; *P* = 0.37
31	χ^2^ = 7.27; *P* = 0.01	χ^2^ = 0.04; *P* = 0.84	χ^2^ = 0.24; *P* = 0.63
32	χ^2^ = 0.66; *P* = 0.42	χ^2^ = 1.93; *P* = 0.16	χ^2^ = 5.07; *P* = 0.02
33	χ^2^ = 2.02; *P* = 0.16	χ^2^ = 0.45; *P* = 0.50	χ^2^ = 0.72 *P* = 0.40
34	χ^2^ = 1.51; *P* = 0.22	χ^2^ = 3.67; *P* = 0.06	χ^2^ = 2.85; *P* = 0.09

The reliability of the scale for items was 0.9915 and 0.7325 for people. Both values indicate the robustness of the items to adequately rank order people on the latent trait.

### Construct validity

Exploratory factorial analysis was performed for the 15-item version of the scale. The KMO test for the final version of the instrument was 0.777, and the Bartlett’s sphericity test was *p* < 0.05, so the exploratory factor analysis was doable. Factor extraction was done by principal component. The exploratory factorial analysis, after Horn’s parallel analysis to compare the explained variance with those obtained in the simulation, obtained one meaningful factor, finding that the own value for the second factor (1.2661) was lower than that obtained in the simulation (1.2669).

### Discriminant validity

We tested two hypotheses. For the first hypothesis, we found that the group of participants informed on the construct (3rd and 4th of Nursing) obtained significantly higher scores on the scale compared to the group of untrained students (Z = − 7.144; *P* < 0.001). The second hypothesis was to evaluate the level of knowledge among the group of untrained participants (1st and 2nd of Nursing degree, all years of the Law Degree). We found that there were no statistically significant differences between the academic courses for the group of untrained participants (Χ^2^ (5) = 9.310; *P* = 0.097).

### Temporal stability

In the test–retest reliability analysis, the ICC between the administrations was 0.81, with a 95% CI [0.692–0.888].

### Descriptive data

The SexContraKnow-Instrument consisted of 15 items with three response options. The instrument showed good estimate values about its validity and reliability to measure the study construct. For the final 15-item questionnaire, the maximum score is 15 points. The mean score was 7.47 (SD = 3.16). Item 2 (‘The male condom is safe if placed just before ejaculation, even if penetration has occurred previously’) had the highest success percentage (93%), while item 14 (‘During sexual intercourse, the vaginal ring can be removed for 2 hours without risk of pregnancy’) had the lowest (15.8%). The most unknown item was 10 (‘The contraceptive skin patch must be applied on the first day of the menstruation cycle’) with 73.9% of respondents selecting the ‘don’t know, don’t answer’ response option (Table 6).

**Table 6:** Descriptive analysis of the items

Item	Percentage of successes	Percentage of mistakes	Percentage of ‘don’t know, no answer’ responses
4	89.9	6.2	3.9
8	93	4.4	2.6
13	74.7	17.6	7.8
15	51.7	20.7	27.6
16	27.6	50.1	22.2
19	53	16.8	30.2
26	62	2.3	35.7
27	37	7.2	55.8
28	62.3	4.9	32.8
29	22	4.1	73.9
30	25.8	11.4	62.8
31	39.3	1.3	59.4
32	43.4	36.4	20.2
33	15.8	37.5	46.8
34	34.9	9	56.1

## Discussion

This research focused on the construction and validation of a tool to measure the level of knowledge about sexuality and contraceptive methods in a university population. The final version of the SexContraKnow-Instrument consists of 15 items (Appendix). The approximate time of completion for the scale is 10 min.

The instrument presents good indicators regarding the validity of the content as a result of the robust construction and review process by the committee of experts, who carried out two reviews prior to the pilot test.

An important aspect of the scale is that it is composed of items with three answer options (‘true’, ‘false’, ‘don’t know/no answer’). With the incorporation of this third response option, we sought to avoid having participants feel obliged to answer the items ‘true’ or ‘false’ in a random way. By adding the answer option ‘don’t know/no answer’, we can identify the topics that participants feel they do not know or have inadequate knowledge about, and this information is useful for planning future educational programmes for the university population.

The objective of this research was to develop and validate an instrument to measure ‘Knowledge about sexuality and contraception; in university students, so items with different levels of difficulty were included to measure all levels of the construct. The very easy items were eliminated because they did not contribute anything to the discriminatory capacity of the scale. To calculate the content validity of scale, a panel of seven experts was used, following the recommendations of Polit and Beck [22]. To determine the validity, it is important to have a sufficient number of experts to improve the reliability of their evaluation. The number of experts is similar to that used in previous studies in which instruments were created concerning similar subject matter to that used in the present scale [41, 42].

Previous studies that evaluated the level of knowledge about sexuality and contraceptive methods in university students found that the main gap in knowledge occurs for items referring to hormonal contraceptive methods, a result that agrees with our research, where the items with a greater percentage of ignorance or errors were those related to the use of the contraceptive pill, vaginal ring or skin patch [18–21].

The items evaluating the level of knowledge about sexuality and the male condom (4, 8, 13 and 26) showed high rates of knowledge with a percentage of correct answers above 62% for all of them. The items referring to hormonal contraceptive methods, divided according to method such as the use of the pill (15, 19, 27 and 28), contraceptive patch (29, 30 and 31) and vaginal ring (32, 33, 34), were the items with the highest percentages of ignorance, with those referring to the use of the contraceptive patch showing the highest percentages. These differences with respect to the level of knowledge found depending on the subject matter of the items can be explained by the contraceptive methods most used by young people; according to the National Survey on Contraception carried out by the Spanish Society of Contraception in 2018, the contraceptive method most used was the male condom, followed by the contraceptive pill, vaginal ring and contraceptive patch [43].

It seems that the use of a contraceptive method causes a person to acquire some level of information about it. Nevertheless, it is necessary to be cautious about this, because as has been observed in previous investigations, the main reasons that lead women to have contrary attitudes concerning hormonal contraception are based on the negative experiences of friends, most of which tend to be caused by incorrect use of the contraception method [13]. More research is needed in the university population to assess the level of knowledge, the main gaps in perceived knowledge and the attitudes of participants towards contraceptive use.

The scale was valid, with good estimated values of discriminative validity showing that it is able to differentiate between participants with high and low levels of knowledge. No DIF was found for any of the items that formed the final version of the scale.

As a result of the exploratory factorial analysis performed to calculate the validity of the construct, the scale presented a one-dimensional structure. During the process of writing the items, following the expert recommendations, we decomposed the items into categories that formed the construct together; however, when the analysis was performed, no evidence was found to support the existence of a substructure or that the construct could be broken down into factors. The one-dimensionality of the construct was considered an advantage for evaluating the level of knowledge, because it offers a direct score through the direct sum of the correct answers.

Finally, the model’s parameters regarding the reliability of the scale for items and people show estimated results of strength with respect to items, although with some limitations for people. This could be due to the size and homogeneity of the sample, so further testing with an increased sample consisting of students from other universities or other degrees is recommended.

The main limitation of this study is that it was not possible to calculate the criteria validity of the instrument because there is no other validated tool that would measure the construct in its entirety in a university population that could serve as a gold standard. Another limitation of our study is that the sample selected for the determination of the psychometric properties of the scale consisted of young university students. The scale has shown acceptable values of reliability and validity for discriminating between trained and untrained, however, we recommend increasing the sample with young people from other universities and degrees and with non-university youth. This limitation means we must be cautious when using the scale, and it must be evaluated psychometrically in non-university youth.

The scale has been validated in Spanish, so we recommend its translation into other languages for future research and so that it can be used in the development of educational programmes.

## Conclusion

In conclusion, the instrument showed good estimated values for its validity and reliability to measure the university students’ level of knowledge about sexuality and contraception. It is a self-administered scale that is easy to fill in on paper, although it can also be completed via the Internet. The instrument has sufficient validity and reliability to recommend its use in future research, although it would be advisable to test it in different universities with students of different degrees and a larger sample. The sample was made up of university students with different levels of ability, and the instrument was able to evaluate their level of ability, so it can be used in a university population aged between 18 and 25 years pursuing different degrees. This scale can be used in future research to evaluate the effect of educational interventions for university youth or as a tool to identify areas of ignorance or misunderstanding for the development of future educational programmes.

## Data Availability

The datasets analysed for this manuscript are available from the corresponding author on reasonable request.

## Appendix

A continuación, encontrarás una serie de afirmaciones sobre sexualidad, embarazo y métodos anticonceptivos. Algunas de las afirmaciones son verdaderas y otras son falsas, indica en cada caso la opción que creas más correcta. Exponemos una tercera opción de respuesta, no sabe/ no contesta (NS/NC). En el caso de desconocer si la afirmación es verdadera o falsa, por favor indícanoslo mediante la opción NS/NC y no contestes al azar.

**Table:**

1- Hay riesgo de embarazo cuando se mantienen relaciones sexuales sin ninguna protección en los dos días previos o posteriores a la ovulación.	V	F	NS/NC
2– El preservativo masculino es seguro si se coloca justo antes de eyacular, aunque previamente haya habido penetración.	V	F	NS/NC
3 – El “método del calendario” (Calcular el periodo fértil para no mantener relaciones sexuales dentro de este periodo) es efectivo para evitar un embarazo.	V	F	NS/NC
4 – Al inicio de la toma de la píldora anticonceptiva, esta es efectiva desde el primer día.	V	F	NS/NC
5– Los métodos anticonceptivos hormonales (por ejemplo, la píldora anticonceptiva o el anillo vaginal) son recomendables para los adolescentes	V	F	NS/NC
6 - Cuando hay un olvido de la toma de la píldora anticonceptiva desde la hora correcta de la toma, se puede tomar sin que haya una pérdida de efectividad siempre que no hayan pasado más de 12 horas desde la hora original	V	F	NS/NC
7 - El “doble método anticonceptivo” consiste en la utilización de manera simultánea de un anticonceptivo de barrera (por ejemplo, preservativo masculino) y uno hormonal (Por ejemplo, píldora anticonceptiva	V	F	NS/NC
8- Si el inicio de la toma de la píldora anticonceptiva es posterior al 5° día del ciclo, es recomendable usar otro método anticonceptivo durante una semana.	V	F	NS/NC
9- La pauta de toma de la píldora anticonceptiva es de una píldora diaria, desde el 1° día del ciclo, durante 21 días con una semana de descanso o durante esta semana tomar 7 pastillas de placebo.	V	F	NS/NC
10 - El parche cutáneo anticonceptivo se debe colocar el primer día del ciclo.	V	F	NS/NC
11 - El remplazo del parche cutáneo anticonceptivo se debe hacer sólo cuando este se desprenda por sí mismo.	V	F	NS/NC
12- El parche cutáneo anticonceptivo se debe colocar preferentemente en el glúteo, zona baja del vientre, zona alta de la espalda o externa de los brazos	V	F	NS/NC
13 - Para la colocación del anillo vaginal es necesario acudir a un médico especialista	V	F	NS/NC
14 -Durante una relación sexual, el anillo vaginal se puede retirar durante 2 horas sin que exista riesgo de embarazo.	V	F	NS/NC
15- El anillo vaginal se debe dejar puesto durante 21 días, dejando posteriormente una semana de descanso.	V	F	NS/NC

Below you will find a series of statements about sexuality, pregnancy and contraceptive methods. Some of the affirmations are true and others are false. Please indicate in each case the option you think is the most correct. There is also a third answer option, ‘Don’t Know/No Answer’; if you do not know whether the statement is true or false, use this third option and do not answer randomly.

**Table:**

1 - There is a risk of pregnancy when you have unprotected sex in the 2 days before or after ovulation.	T	F	Don’t Know/No Answer
2 - The male condom is safe if placed just before ejaculation, even if penetration has occurred previously.	T	F	Don’t Know/ No Answer
3 - The ‘calendar method’ (calculating the fertile period so as to not have sexual intercourse within this period) is effective in preventing pregnancy.	T	F	Don’t Know/ No Answer
4 - When you start taking the birth control pill, it is effective from day one.	T	F	Don’t Know/ No Answer
5 - Hormonal contraceptive methods of birth control (for example, the birth control pill or vaginal ring) are recommended for adolescents.	T	F	Don’t Know/ No Answer
6 - When the contraceptive pill is not taken at the correct time due to forgetfulness, the pill can be taken without loss of effectiveness as long as no more than 12 h have passed since the correct time.	T	F	Don’t Know/ No Answer
7 - The ‘dual contraceptive method’ consists of the simultaneous use of a barrier contraceptive method (e.g. male condom) and a hormonal contraceptive method (e.g. contraceptive pill).	T	F	Don’t Know/ No Answer
8 - If the contraceptive pill is started after the 5th day of the menstruation cycle, use of another contraceptive method for 1 week is recommended.	T	F	Don’t Know/ No Answer
9 - The regimen for taking the contraceptive pill is one pill per day for 21 days starting from the 1st day of the cycle, followed by a week of rest or use of 7 placebo pills during this rest period.	T	F	Don’t Know/ No Answer
10 - The contraceptive skin patch must be applied on the first day of the menstruation cycle.	T	F	Don’t Know/ No Answer
11 - The birth control skin patch should be replaced only when the patch detaches itself.	T	F	Don’t Know/ No Answer
12 - The contraceptive skin patch should be placed on the buttocks, lower abdomen, upper back or outer arms.	T	F	Don’t Know/ No Answer
13 - It is necessary to see a specialist for placement of a vaginal ring.	T	F	Don’t Know/ No Answer
14 - The vaginal ring can be removed for 2 h during sexual intercourse without risk of pregnancy.	T	F	Don’t Know/ No Answer
15 - The vaginal ring should be left in place for 21 days, followed by a week of rest.	T	F	Don’t Know/ No Answer

## Abbreviations

CI: Confidence interval
ICC: Intraclass correlation coefficient
KMO: Kaiser–Meyer–Olkin
SD: Standard deviation
SexContraKnow-Instrument: Sexuality and Contraceptive Knowledge Instrument
